# Orthodontic Intervention to Impacted and Transposed Lower Canines

**DOI:** 10.1155/2017/4105713

**Published:** 2017-04-30

**Authors:** Nihat Kılıç, Hüsamettin Oktay

**Affiliations:** ^1^Department of Orthodontics, Faculty of Dentistry, Atatürk University, Erzurum, Turkey; ^2^Department of Orthodontics, School of Dentistry, Istanbul Medipol University, Istanbul, Turkey

## Abstract

Impacted and transposed teeth cause serious difficulties in tooth eruption and movement as well as esthetic and functional outcomes. Proper treatment planning including good biomechanical control is essential in order to avoid side effects during traction and aligning of the impacted and/or transposed teeth. The purpose of the present study was to present a successfully treated female patient having transposed and impacted lower canines by means of a modified lingual arch and fixed orthodontic appliance. A female patient aged 13 years and 9 months presented to the orthodontic department with a chief compliant of bilateral spacing and missing teeth in mandibular dentition. After leveling and creating sufficient space in the mandibular arch for the canines, a modified lingual arch was cemented to the mandibular first molars. The lingual arch had two hooks extending to the distobuccal areas of the canine spaces. Elastic chains were applied between the hooks on the lingual arch and the ligatures tied to the attachments on the canine crowns. The light forces generated by elastic materials caused impacted canines to erupt and tend towards their own spaces in the dental arch. As a result, impacted and transposed lower canines were properly positioned in their spaces, and the treatment results were stable during the retention period.

## 1. Introduction

Impaction refers to a failure of a tooth to emerge into the dental arch, usually due to either space deficiencies or presence of an entity blocking its path of eruption [1]. Tooth transposition is the positional interchange of two adjacent teeth [2]. Both impacted and transposed teeth cause serious difficulties in tooth eruption and movement as well as esthetic and functional outcomes.

Impaction of mandibular canines is encountered less frequently [3], and its incidence is 0.35% [4]. According to Kerr [5], mandibular canine impaction is twentyfold less than the maxillary impaction of these teeth.

A number of reasons may be responsible for canine impactions such as mechanical obstruction of eruption pathway and/or insufficient space in dental arch due to skeletal discrepancy (micrognathia inferior), premature loss of deciduous teeth, or tooth size discrepancy [4].

The purpose of the present study was to present a successfully treated female patient having transposed and impacted lower canines by means of a modified lingual arch and fixed orthodontic appliance.

## 2. Diagnosis and Treatment Planning

**Figure 1:**
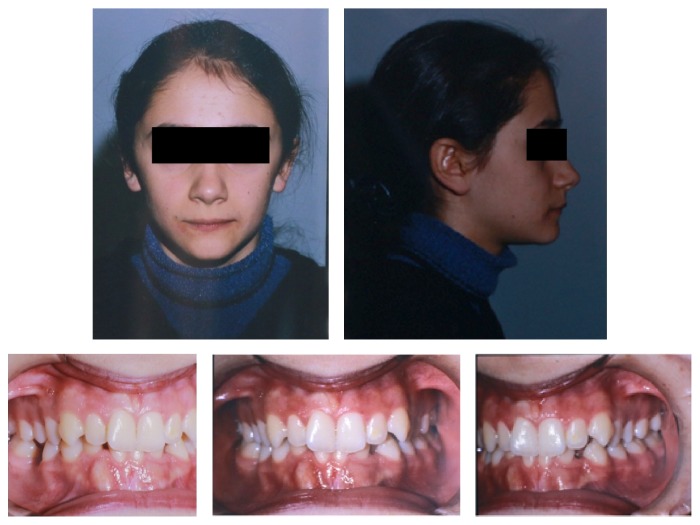
Pretreatment extraoral and intraoral photographs of the patient.

**Figure 2:**
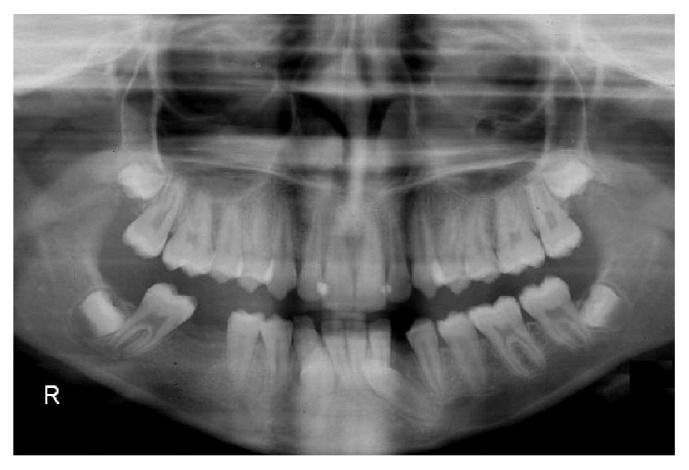
Pretreatment orthopantomograph.

A female patient aged 13 years and 9 months presented to the Orthodontic Department of Dentistry School (Atatürk University, Erzurum, Turkey) with a chief complaint of bilateral spacing and missing teeth in mandibular dentition. She was in good health. Clinical examination revealed that the patient had an acceptable facial profile, end-to-end Class II molar relationship, mild crowding in upper and lower dentitions, normal overjet (3 mm), and increased overbite (5 mm) (Figure 1). Cephalometric analysis showed a Class I skeletal relationship, normal vertical dimensions, slightly proclined lower incisors and retroclined upper incisors, and normal positioned lips (Table 1). Orthopantomograph of the patient revealed that the lower right canine was transposed and impacted in an upright position between the roots of right central and lateral incisors and the left canine was horizontally impacted at the root levels of the left central and lateral incisors. This radiograph also showed that the lower right first molar was extracted previously (Figure 2).

### 2.1. Treatment Objectives

In order to obtain a well-balanced occlusion and good esthetic results, the following treatment objectives for this patient were planned:

- Elimination of spacing and crowding in both arches
- Creating adequate spaces in the mandibular arch to accommodate the canine teeth
- Surgically exposing the canines in order to bond orthodontic attachments
- Applying an orthodontic traction by light forces to bring the impacted and transposed canines into the dental arch

### 2.2. Treatment Progress

**Figure 3:**
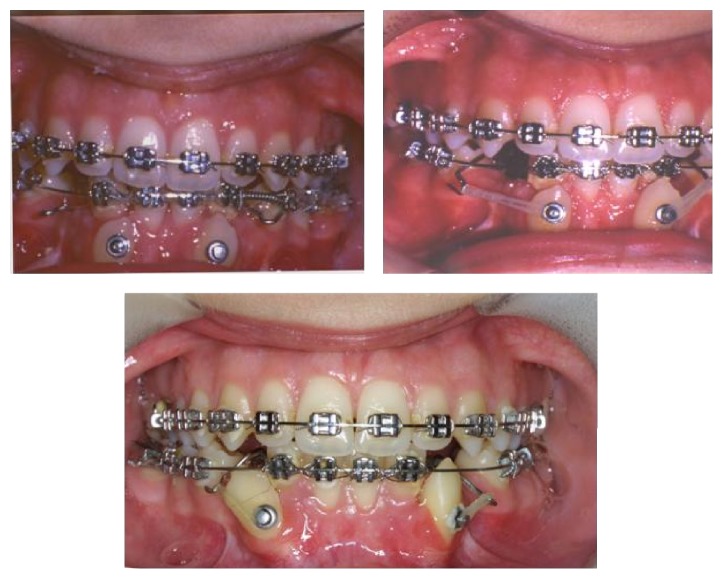
Elastic traction in different times of the treatment.

Under local anesthesia, the right and left mandibular canines were surgically exposed, and bonded attachments were placed on the labial surfaces of them. Then, mucosal flap was sutured so that it completely covered the impacted teeth and attachments. Multibracket fixed appliances were placed to the maxillary and mandibular teeth. Treatment was begun with a phase of leveling and aligning of the teeth in both arches. After leveling and creating sufficient space in the mandibular arch for the canines, a modified lingual arch was cemented to the mandibular first molars. The lingual arch had two hooks extending to the distobuccal areas of the canine spaces. Elastic chains were applied between the hooks on the lingual arch and the ligatures tied to the attachments on the canine crowns. Direction of the force was adjusted by changing the location of the hooks so that the canines moved distally and occlusally without touching the neighboring teeth. The light forces generated by elastic materials caused impacted canines to erupt and to tend towards their own spaces in the dental arch. In order to obtain a better occlusal relationship for the canines, the attachments on their crowns were repositioned towards the gingival side, and then they were replaced with brackets (Figure 3). After the canines were positioned in their spaces, the hooks of the lingual arch were cut, and ideal occlusal relationship was established. Unfortunately, lower left first premolar was lost due to caries and periapical inflammation during aligning and leveling phase. Total treatment time was 4 years and 3 months, and removable retainers were used for two years to maintain the obtained stable results.

### 2.3. Treatment Results

**Figure 4:**
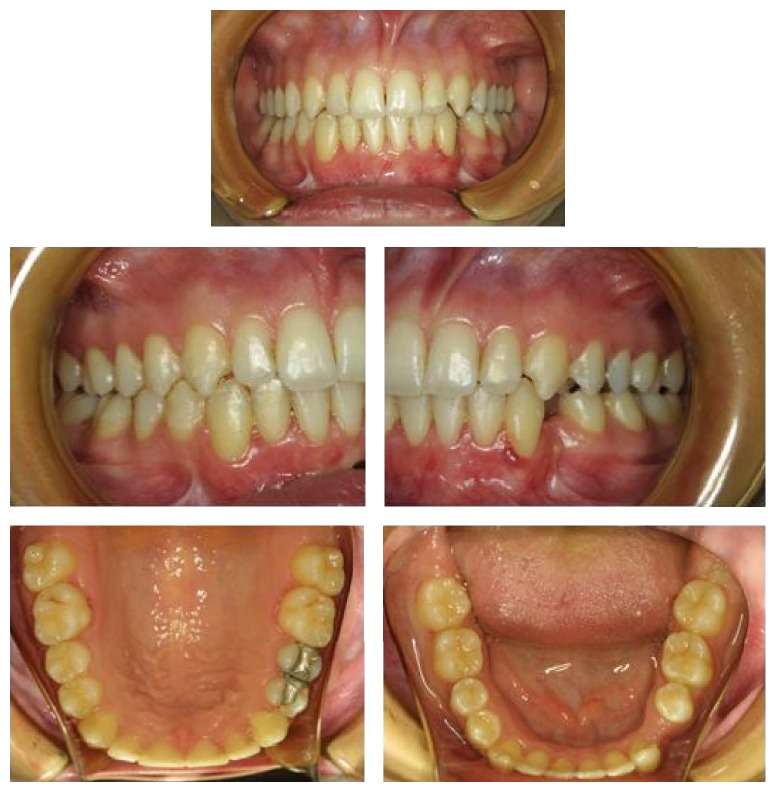
Posttreatment intraoral photographs of the patient.

**Figure 5:**
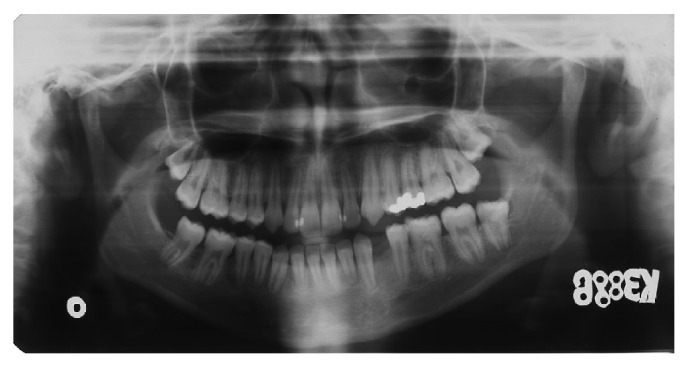
Posttreatment orthopantomograph.

**Figure 6:**
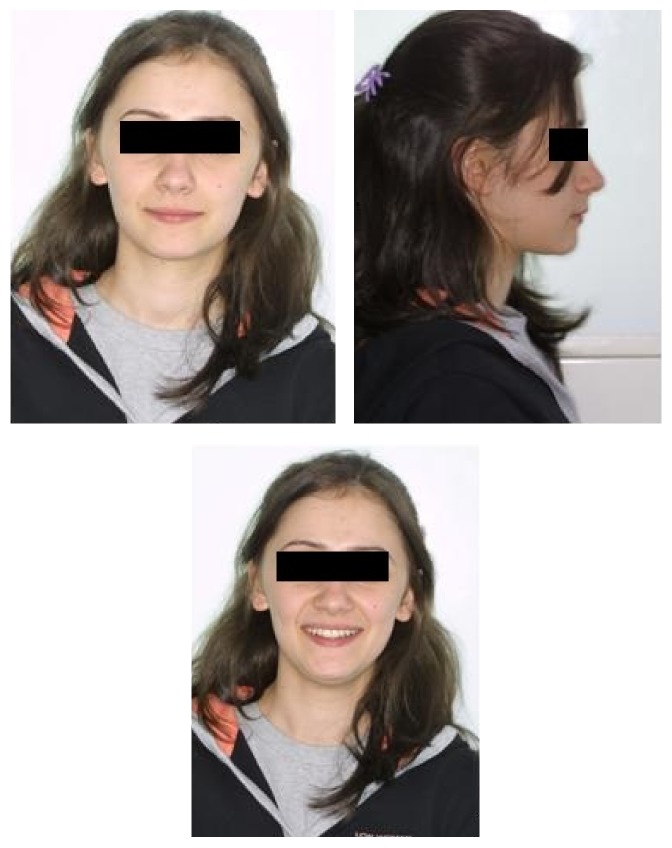
Posttreatment extraoral photographs of the patient.

Class I molar and canine relationships were established with a normal overjet and overbite after treatment. The mandibular canines were brought to their own spaces, without any discomfort and side effect such as root resorption, alveolar destruction, and gingival recession, and they were properly positioned in the dental arch (Figures 4 and 5). Periodontal tissues were healthy during the treatment and retention periods. The patient showed minimal growth increments during these periods with a good final profile (Figure 6).

## 3. Discussion

In the orthodontic management of impacted and/or transposed canines, the clinician has to make certain decisions regarding fixed appliance versus removable appliance treatment, one-arch versus two-arch treatment, and canine versus first premolar extraction [6]. Factors such as damage to the adjacent structures and periodontal health of the impacted canines after treatment should be considered [7]. Erupting, leveling, and aligning of the impacted teeth, especially horizontally impacted canines, may compromise difficult clinical procedures to provide optimal treatment options with the most stable and favorable outcome [6].

Orthodontic movement of the impacted canine after surgical exposure is the most common treatment approach [8], and different surgical-orthodontic techniques have been described in the literature [6, 9–12]. Spencer [11] used canine uprighting hooks embedded to the progressive bionator appliance and applied traction and uprighting forces with elastics in order to erupt a horizontally impacted lower canine. Treatment time of this case was approximately four and a half years.

Bishara [6] suggested six treatment options in impacted canine cases: no treatment if the patient does not desire; autotransplantation of the canine; extraction of the impacted canine and movement of the first premolar in its space; extraction of the canine and posterior segmental osteotomy to close the canine space; prosthetic replacement of the canine; surgical exposure of the canine and orthodontic treatment to bring the tooth into the dental arch. According to the authors, the latest treatment option is obviously the most desirable approach.

In the present case report, transposed and horizontally impacted lower canines were surgically exposed and brought into proper occlusion with surgical-orthodontic management. With the introduction of new orthodontic materials such as elastic threads and elastomeric chains, orthodontist has greater control of force magnitude and direction. Regardless of the material used, direction of the initial force should be to move the tooth away from the neighboring teeth to avoid their injury [6]. In the present case, lower canines were brought into their own places without any injury to the neighboring teeth and structures (Figures 4 and 5).

As a result, impacted and transposed lower canines were properly positioned in their spaces, and the treatment results were stable during the retention period.

We want to draw attention to an unpleasant condition in this case. Lower left first premolar of the patient was lost due to caries and periapical inflammation during aligning and leveling phase of the orthodontic treatment, although a strict oral hygiene education was given to the patient before treatment. Decalcification, caries, and periodontal problems associated with orthodontic treatment have been reported in literature [13]. Shannon stated that orthodontic patients were under a great risk of decalcification or caries, since oral hygiene problems occur inevitably when the fixed appliances are worn [14]. The banded and bonded orthodontic appliances increase the number of sites conducive to plaque retention, and, as a result, oral hygiene becomes more difficult. This encourages lower pH value of dental plaque adjacent to orthodontic brackets, which hinders remineralization and can lead to decalcification of enamel [15]. This fact we faced revealed the importance of collaboration between the orthodontist and general dentist to prevent the unwanted clinical situations such as decalcifications, caries, and periodontal problems.

## Figures and Tables

**Table 1:** Cephalometric measurements at pre- and posttreatment.

	Norm	Pretreatment	Posttreatment
SNA°	82	79	79
SNB°	80	76	76.5
ANB°	2	3	2.5
SN-GoMe°	32	38	37.5
IMPA°	95	108	110
U1-NA (mm)	4.3	2.5	3
U1-NA°	22.8	15	17
L1-NB (mm)	4	4	5
L1-NB°	25.3	22	28

°: degree.
